# Exploring lie frequency and emotional experiences of deceptive decision-making in autistic adults

**DOI:** 10.1177/13623613251315892

**Published:** 2025-03-03

**Authors:** Tiegan Blackhurst, Lara Warmelink, Amanda Roestorf, Calum Hartley

**Affiliations:** 1 Lancaster University, UK; 2 Autistica, UK

**Keywords:** autism, emotion, lie frequency, motivation, orientation

## Abstract

Deception is a multi-faceted social behaviour that is pervasive in human communication. Due to differences in social communication and experiences, autistic and non-autistic adults may contrast in how they respond to situations that elicit deceptive decision-making. This study examined whether autistic and non-autistic adults differed in their general lie frequency, their inclination to produce different lie types, and their emotional experiences of lying. Fifty-eight non-autistic and fifty-six autistic university students matched on age and gender completed self-report measures of their general lying patterns, how often they lied in the past 24 hours, and whether they would lie across hypothetical scenarios with differing beneficiaries (self, other, group) and motivations (protective, beneficial). The groups did not significantly differ in their general lying behaviour or frequency of lies told over 24 hours. Yet, autistic adults indicated that they would be significantly less likely to lie in group scenarios and would experience increased difficulty, more guilt, and greater concerns about their believability when lying. These results advance theoretical understanding by suggesting that autistic adults’ deceptive decision-making may be context-dependent. Future research may benefit from examining autistic deception across numerous social situations as more general lie frequency measures may be insensitive to nuanced population differences.

**Lay Abstract:**

Lying, a universal social behaviour, is frequent in everyday communication. Due to differences in social communication and experiences, autistic and non-autistic adults may react differently in situations where they must decide whether to lie or tell the truth. We investigated whether autistic and non-autistic adults differ in their general lying behaviour (e.g. how often they lie) and their likelihood of lying in a range of hypothetical social scenarios with different motivations (why people lie – to benefit or protect) and orientations (who people lie for; themselves, other, a group). We also examined participants’ emotional experiences of lying and truth-telling. We found that autistic and non-autistic adults’ general lying frequencies and emotional experiences were similar. However, the social scenario responses revealed that autistic adults would be less likely to lie to benefit or protect a social group they are part of. Moreover, autistic adults indicated that they would find lying more difficult across all social scenarios, experience more guilt, and would be less confident that their lie would be believed. This research highlights how autistic adults’ lying may be context-dependent and considers how a reduction in the likelihood of lying for their social group could increase strain on autistic adults’ social relationships.

## Introduction

Choosing to lie is a complex process that involves considering why one needs to lie, who one is lying for, and the medium required to deliver the lie (e.g. face-to-face, computer-mediated; Cantarero et al., 2018). These deception processes have been researched extensively in non-autistic populations (Serota & Levine, 2015; Smith et al., 2014), but deceptive decision-making may differ across cultures (e.g. western and eastern cultures) and neurodiverse populations (Ennis et al., 2008; Griffin & Bender, 2019). For example, individuals with Autism Spectrum Condition may experience deceptive decision-making differently due to difficulties with social communication and social experience (American Psychiatric Association [APA], 2013). Indeed, developmental evidence suggests that autistic children often choose to lie less frequently than non-autistic children (Talwar et al., 2012). However, research has yet to comprehensively investigate deceptive decision-making in autistic adults, despite social demands and relationships becoming significantly more complex beyond childhood (Walczyk & Fargerson, 2019; Wood et al., 2018). Exploring if, when, and why autistic adults lie is necessary to advance understanding of how their deception may vary across different social situations and how their subjective experiences of deceptive-decision making may differ from non-autistic adults’.

### Lie frequency

DePaulo et al. (1996) reported that most non-autistic adults lie once or twice per day, although Serota et al. (2010) suggest that average lie prevalence rates are inflated by prolific liars responsible for the majority of lies. Data from different countries (Park et al., 2021), mediums (Smith et al., 2014) and age groups (Levine et al., 2013; Serota and Levine, 2015) support this theory. Serota et al. (2010) also report large differences in lie frequency across the lifespan; specifically, prolific lying decreases with age. However, most lie prevalence research assesses one type of lie in one situation (Serota et al., 2010). Thus, extant data are often restricted by context and do not capture the variety of motivations and orientations that people encounter in deceptive decision-making (e.g. one may not lie to protect themselves, but may lie to protect their friend). As such, it is unclear whether current conclusions regarding lie frequency generalise accurately across different deceptive situations.

The majority of research investigating lie frequency in autism has been conducted with children (e.g. Baron-Cohen, 1992; Li et al., 2011; Talwar et al., 2012) and has produced conflicting results. Some studies indicate that autistic children lie less frequently than non-autistic children (Talwar et al., 2012) while others suggest there are no differences in lie frequency across populations (Li et al., 2011). To date, just two studies have focused on lie frequency in autistic adults. In the study by Van Tiel et al. (2021), participants played a computer game in which they had to lie to win against a computerised opponent. The results showed that autistic adults were just as likely to deceive the computer as non-autistic adults. Bagnall et al. (2023) explored general inclinations to lie using ‘Lying in Everyday Situations’ statements (e.g. ‘I lie for revenge’; Hart et al., 2019). Their results revealed that autistic adults reported lying as often as non-autistic adults, even when believing themselves to be poorer liars. These studies suggest that autistic adults do not differ from non-autistic adults in their lying frequency. However, these methodologies do not represent the social pressures experienced in real life, nor do they provide insight into whether autistic and non-autistic adults’ lying decisions differ across social contexts. To understand the relationship between autistic adults’ deceptive decision-making and social context, it is necessary to examine lie frequency across different situations and draw comparisons with more general measures of lie frequency utilised in prior research.

As many autistic individuals experience difficulties with social interaction across their lifespan, it is plausible that differences in lie frequency may emerge when probing specific kinds of socially motivated deception (DePaulo et al., 1996; Runcharoen, 2014). From 2 years of age, non-autistic children learn how to deceive and what deception looks like via social interactions and modelling others’ deceptive behaviour (Engarhos et al., 2020; Evans & Lee, 2013). However, many autistic children spend less time engaged in communicative interactions than non-autistic peers, reducing their opportunity to observe deceptive behaviour (Goddard & Cook, 2022). For some autistic individuals, this trend of limited social interaction continues into adulthood, with difficulties in social communication reducing the duration, quantity and quality of their social experiences (Seltzer et al., 2004). These differences in social experience may inhibit autistic individuals from inferring acceptable and expected patterns of deception across diverse social situations (e.g. recognising when telling the truth would cause more harm than engaging in prosocial deception, such as telling a ‘white lie’ about liking a friend’s new hair style; Jaarsma et al., 2012). Consequently, autistic individuals may choose to engage in deception less frequently than non-autistic peers in particular social situations that feel unfamiliar. If autistic adults are less likely to lie in social situations where they are beneficial or expected, this may negatively impact their ability to form and maintain relationships (Lupoli et al., 2017).

### Lie type ~ motivation and orientation

**Figure 1.**
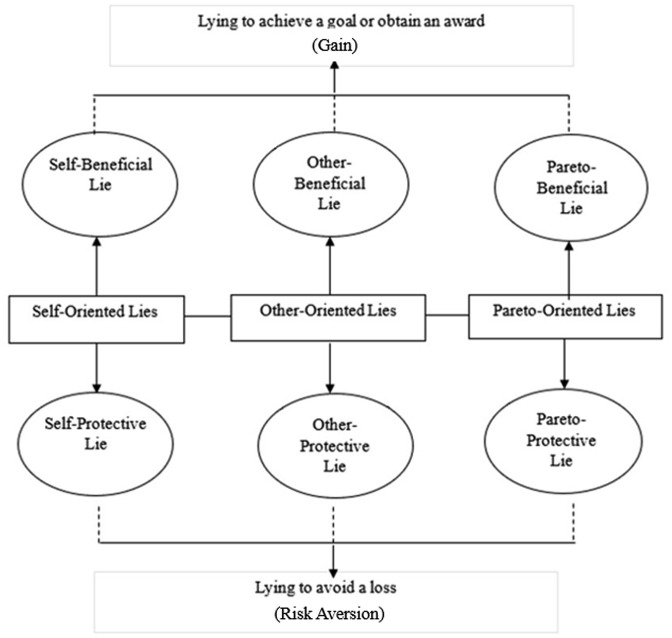
The typology of everyday lies by Cantarero et al. (2018).

Cantarero et al. (2018) proposed a typology of everyday lies with distinct motivations and orientations. ‘Motivation’ refers to why the lie is being told, including protective lies told to avoid negative consequences and beneficial lies told to achieve goals or gains. ‘Orientation’ refers to the lie’s beneficiary: the self, another or group that the self belongs to (pareto-oriented lies; see Figure 1). Self-oriented lies benefit or protect oneself (e.g. lying about why you are late for work), other-oriented lies benefit/protect another person (e.g. lying to avoid harming your best friend’s feelings) and pareto-oriented lies benefit/protect a social group (e.g. lying to gain a positive outcome for your friendship group). Cantarero et al. (2018) found that protective lies were told more frequently than beneficial lies, aligning with the earlier proposal by Schmidt and Traub (2002) that people experience greater negative emotional reactions following a loss than positive emotional reactions following a gain (i.e. they display loss aversion). Cantarero et al. also found that self-oriented lies were told more frequently than other- and pareto-oriented lies, suggesting that many individuals exhibit self-serving biases (see Schechtman, 2002). However, Whitty and Carville (2008) found that although individuals tell more self-oriented lies to strangers, they tell more other-oriented lies to close acquaintances. The reason for this may be twofold: (1) people close to us know more about us, hence self-oriented lies may be easily identifiable and (2) people tell more prosocial lies to people they care about to protect their feelings (Ennis et al., 2008). Therefore, the identity of recipients influences the frequency and types of lies told by non-autistic adults.

Regarding motivation, Li et al. (2011) reported that both non-autistic and autistic children lie to conceal transgressions and protect others from harm. In addition, Gosling and Moutier (2018) report that autistic adults are as risk-averse as non-autistic adults, suggesting that both populations may be more inclined to tell protective lies to avoid experiencing negative consequences (i.e. avoid loss) than beneficial lies to receive a gain. Concerning orientation, self-oriented lies rely more heavily on the individual’s own thoughts and perspectives as they are intended to advantage or protect the liars’ own interests (Kashy & DePaulo, 1996). By contrast, other- and pareto-oriented lies require taking the perspective of the person being lied for (who may or may not be the same person as the person being lied to) in order to serve not just the liar but another person or group (Cantarero et al., 2018). Awareness of others’ mental states (i.e. Theory of Mind) is often believed to be a pre-requisite to deceit. To successfully lie, one must consider what the recipient knows to be true and anticipate their thoughts and emotions when formulating an appropriate response (e.g. a friend will be upset if I say that I dislike their new coat, so I choose to lie; Sip et al., 2008). However, recent meta-analytic evidence suggests that the association between Theory of Mind and lying in non-autistic populations may be moderated by additional variables (e.g. facet of lying) and that Theory of Mind is only weakly related to spontaneous and instigated lying (Lee & Imuta, 2021). These findings suggest that, even if individuals have the perspective-taking abilities required to lie, whether people choose to engage in deceit may be determined by other factors such as lie valence (whether the lie is antisocial or prosocial) and social experience (e.g. whether a person has experienced the consequences of truthfulness or lie-telling in a given situation; Talwar & Lee, 2011).

Whether someone decides to tell a pareto lie may also be influenced by their experiences of social group membership. Autistic adults who experience difficulties with social communication and interaction from a young age (Baron-Cohen, 2008; Locke et al., 2010) may be less likely to seek and/or gain group membership. Consequently, autistic adults may tell fewer pareto-oriented lies than non-autistic adults in larger group situations due to their relative lack of exposure to such interactions (Baron-Cohen, 2008). Although autistic adults may have less experience interacting with non-autistic adults in group situations (Dean et al., 2014), they do seek social contact with other autistic adults and often report having at least one strong friendship (Bauminger et al., 2008; Crompton et al., 2020). As such, autistic and non-autistic adults may show similarities in lie frequency across self- and other-oriented situations.

### Emotional experience of lying

Telling different types of lies may elicit contrasting emotional responses. When telling other-oriented lies to benefit or protect someone else, non-autistic adults may experience positive emotions (e.g. relief) as their deceit may have favourable outcomes (Smetana & Wainryb, 2021). However, when lying to protect or aid oneself, non-autistic adults may experience negative emotions (e.g. guilt) as society deems such lies to be immoral (Vrij et al., 2011). Feelings of guilt may also arise if one tells the truth when electing to deceive may have caused less harm. Thus, the anticipated outcome of a lie may elicit stronger emotional reactions in communicators than the act of deception itself (E. E. Levine, 2022).

Little is known about how autistic adults experience lying, although anecdotes from autobiographies suggest that lying may be associated with adverse emotional reactions. Grandin (2006, p. 156) stated ‘*I become extremely anxious when I have to tell a little white lie*’ and Birch (2003, p. 121) reported that they found lying ‘*very painful*’. Such intense emotions may deter autistic adults from lying and indicate that they may be less likely to experience positive emotions when deceiving. Crucially, such emotional experiences may inhibit autistic adults from engaging in prosocial deception, which is considered to be an important developmental milestone (Li et al., 2011). Prosocial lying positively contributes to maintaining adaptive social relationships by facilitating conflict avoidance, protecting social partners from emotional harm and increasing trust (E. E. Levine & Lupoli, 2022; E. E. Levine & Schweitzer, 2015). If autistic adults do not engage in prosocial deception, this may increase strain on their relationships as their honesty may lead to conflict with social partners, exacerbating communication difficulties and leading to feelings of isolation and anxiety (Jaarsma et al., 2012; E. E. Levine & Schweitzer, 2015).

### This study

This study is the first to investigate differences between autistic and non-autistic adults in lie frequency and experiences of lying across social contexts. Participants first reported how often they lied in general, then how often they had lied in the past 24 hours, to who, and using which medium (Serota & Levine, 2015). Then, participants reported whether they would lie or tell the truth, and how they would feel doing so, across a range of hypothetical lying scenarios with different motivations (beneficial, protective) and orientations (self, other, pareto-oriented). Following Van Tiel et al. (2021) and Bagnall et al. (2023), we predicted that there would be no differences between autistic and non-autistic adults’ general lie frequency or lie frequency over the past 24 hours. However, due to differences in social experience and cognition (APA, 2013), we predicted that autistic adults would lie less frequently than non-autistic adults when responding to certain hypothetical social scenarios. Specifically, we expected that autistic adults would be less inclined to tell pareto- and other-oriented lies than non-autistic adults, but populations would not differ in their likelihood of generally telling lies with beneficial or protective motivations. Finally, we anticipated that autistic adults would find lying more difficult and emotionally demanding than non-autistic adults. The results from this study advance theoretical understanding of deception in autistic adulthood, particularly our knowledge regarding the contexts in which autistic adults may choose to lie and their subjective experiences of deceptive decision-making.

## Methods

### Participants

One-hundred and thirty-six participants volunteered to participate by responding to adverts distributed via a research participation system, posters and university services. From this sample, 22 were excluded as: English was not their native language (4), they were seeking an autism spectrum disorder (ASD) diagnosis (i.e. they could not be assigned to the ASD or non-autistic sample with certainty, 10), their age was a statistical outlier at least 2 *SD*s over their group mean (5), or missing data (3). The final sample consisted of 114 participants including 58 non-autistic adults (12 male, 40 female, 6 other/third gender, *M age* = 19.40 years, *SD* = 1.36) and 56 autistic adults (10 male, 35 female, 11 other/third gender, *M* age = 19.73 years, *SD* = 1.46, *M* age of diagnosis = 15.92 years). Socioeconomic status and ethnicity data were not recorded. All participants were registered students at the University of Lancaster at the time of participation (see Table 1). Full anonymised data are openly accessible (see Supplementary Materials).

**Table 1.** Characteristics of autistic and non-autistic participants (*SD* and ranges in parentheses).

	Population		Group comparison *t* test (*p*)
	ASD	NA	
*N*	56	58	
Gender	10 male, 35 female, 11 third/other gender	12 male, 40 female, 6 other/third gender	
Age (*M* years)	19.73 (1.46; 18–24)	19.40 (1.36; 18–23)	1.27 (0.207)
D-KEF towers task raw score (*M*)	18.39 (3.42; 10–27)	18.07 (3.24; 11–26)	0.52 (0.605)
Performance IQ raw score (*M*)	71.63 (12.15; 32–90)	68.48 (13.18; 41–92)	1.32 (0.188)
Verbal IQ raw score (*M*)	64.16 (7.46; 43–82)	57.40 (9.39; 37–80)	4.27 (< 0.001)
AQ raw score (*M*)	35.93 (7.21, 20–46)	20.17 (7.88, 6–41)	11.14 (< 0.001)

ASD: autism spectrum disorder; NA: non-autistic; DKEF: Delis and Kaplan Executive Functioning Tower Task; AQ: autism quotient.

Our samples of autistic and non-autistic adults did not significantly differ on age (*t*(112) = 1.27, *p* = 0.207), gender (χ^2^ (112) = 2.48, *p* = 0.478), executive functioning measured by the D-KEF Towers Task (*t*(112) = 0.52, *p* = 0.605, Delis et al., 2001), or performance intelligence quotient (IQ) measured by the block design and matrix reasoning sub-tests of the Abbreviated Weschler Intelligence Scale (A-WAIS; Wechsler, 2008; *t*(112) = 1.32, *p* = 0.188). However, autistic adults scored significantly higher on verbal intelligence measured by vocabulary and similarities sub-tests of the A-WAIS (*t*(112) = 4.27, *p* < 0.001, *d* = 0.79). Autistic adults also reported significantly more autistic traits on the Autism Quotient (AQ; Baron-Cohen et al., 2001) compared to non-autistic adults (*t*(112) = 11.14, *p* < 0.001, *d* = 2.09).

Participants received either university course credits or £30 as remuneration. Participants provided informed consent prior to their involvement in the study and all procedures were in accordance with ethical standards of institutional and national research committees. Participants completed this study as part of a larger project, including a range of tasks not analysed here (for more details, see the pre-registration *https://osf.io/b29xu*).

### Materials and covariates

#### Eligibility questionnaire

All interested participants completed an eligibility questionnaire in which they provided their age, gender, autism diagnostic status and indicated any other neurodevelopmental conditions.

#### Diagnostic information questionnaire

Autistic participants stated their age at diagnosis and which interventions (if any) they had experienced.

#### General lie frequency

Participants reported how often they lied in general (e.g. once a week, once a month; see Supplementary Materials p.2), how difficult they found lying (e.g. 1 = not difficult at all, 7 = extremely difficult), how guilty they felt when lying (e.g. 1 = no guilt at all, 7 = extremely guilty) and how often they were believed (e.g. 1 = never, 7 = always; Warmelink & McLatchie, in preparation).

#### Serota grid

Participants reported how often they had lied in the past 24 hours, to who (e.g. family, friend; see Supplementary Materials, p. 2), using which mediums (e.g. face-to-face, computer-mediated; Serota et al., 2010).

#### Lying scenario questionnaire for students

Participants were asked whether they would lie in response to 12 hypothetical scenarios reflecting different orientations and motivations (8 from the original questionnaire and 4 pareto-oriented scenarios created for this study; see Supplementary Materials, Table 1; adapted from Warmelink & McLatchie, in preparation). The hypothetical context-specific lying scenarios were created to reflect the diverse and nuanced situations in which people face lying decisions in naturalistic settings. As such, the scenarios naturally vary in terms of their social acceptability, level of severity and level of difficulty.

If participants stated they would lie in response to a scenario, they were asked how guilty they would feel (e.g. 1 = no guilt at all, 7 = extremely guilty), their confidence in being believed (e.g. 1 = I’m sure I wouldn’t be, 7 = I’m sure I would be) and how difficult they would find lying (e.g. 1 = not difficult at all, 7 = extremely difficult). If participants indicated that they would not lie, they were asked why and how they would feel telling the truth (e.g. whether they would experience a range of emotions, such as happy and relaxed scoring (1) if they would or (0) if they would not; see Supplementary Materials p.6).

### Procedure

Participants who met our eligibility criteria were invited to a research laboratory at Lancaster University. First, autistic participants completed a diagnostic information questionnaire. As this study was part of a larger project, there were three different task orders; participants completed the questionnaires reported here as their first, fifth or seventh task. On average, all questionnaires took 15–20 min to complete.

### Community involvement

Members of the autism community were not involved in the design or implementation of this study. However, Autistica contributed to interpreting the study’s findings through discussion with their scientific research team, which includes autistic individuals.

## Results

### Lying in general

#### Correlations between measures

Following standard practise in this field (Ennis et al., 2008; Serota et al., 2022; Verigin et al., 2019; Warmelink & McLatchie, in preparation), correlational analyses were conducted to assess consistency between lying frequency measures. A Spearman’s Rank correlation indicated a significant positive correlation between responses on the General Lie Frequency questionnaire and Serota Grid *r*(112) = 0.49, *p* < 0.001, with higher levels of general lying associated with increased frequencies of lying in the past 24 hours. A Pearson’s Product–Moment correlation revealed that the number of lies participants reported on the Lie Scenario Questionnaire did not significantly correlate with participants’ general lying *r*(112) = 0.179, *p* = 0.057, or lying in the past 24 hours, *r*(112) = 0.17, *p* = 0.072. These results suggest that, across non-autistic and autistic adults, self-reported general lying behaviour does not strongly predict deceptive decision-making in specific social situations.

#### Lying in general and over the last 24 hours

The general lie frequency data were not normally distributed (Shapiro–Wilks < 0.05 for all variables). Therefore, Wilcoxon Mann–Whitney *U*-tests were used to examine differences in lying frequency and experiences of lying between autistic and non-autistic participants (see Table 2). Autistic and non-autistic adults did not significantly differ on general lie frequency, which medium they used or who they lied to. However, autistic adults reported feeling significantly more guilt, feeling less confidence in their likelihood of being believed and increased difficulty when lying compared to non-autistic adults.

**Table 2.** Differences between autistic (ASD) and non-autistic (NA) adults on general lie frequency, lie medium, lie recipient and emotional experiences of lying.

	ASD	NA			
	*M* (*SD*)	*M* (*SD*)	*U*	*p*	*η* ^2^
Lie frequency					
General lying	4.57 (1.68)	5.07 (1.93)	1361	0.130	0.02
Lies told in the past 24 h	1.84 (2.21)	2.15 (2.25)	1410	0.214	0.01
Lie medium (last 24 h)					
Lies told face-to-face	0.93 (1.29)	1.27 (1.66)	1384	0.149	0.02
Lies told via phone/email/writing	1.29 (1.27)	1.66 (1.44)	1618	0.973	0.00
Lie recipient (last 24 h)					
Lies told to family	0.45 (0.81)	0.48 (0.99)	1633	0.950	0.00
Lies told to friends	0.79 (1.45)	0.91 (1.05)	1390	0.149	0.02
Lies told to acquaintances	0.43 (0.97)	0.40 (0.75)	1589	0.800	0.00
Lies told to strangers	0.05 (0.23)	0.19 (0.48)	1456	0.074	0.01
Lies told to a large group	0.02 (0.13)	0.05 (0.29)	1596	0.581	0.00
Experience of lying					
Guilt (1–7)	3.91 (1.67)	3.31 (1.37)	2036	0.017	0.05
Believability (1–7)	3.5 (1.28)	4.24 (1.23)	1075	0.001	0.09
Difficulty (1–7)	3.77 (1.46)	3.05 (1.30)	2076	0.009	0.06

The general lying mean indicates participants lied, on average, one to four times a week (see Supplementary Materials for a full code break-down).

### Lying and truth-telling in the lying scenario questionnaire

#### Mixed-effects models

The lying scenario data were analysed via generalised linear mixed-effects and cumulative link models using *glmer* and *clmm* functions from the *lme4* and ordinal packages in R (Version /2023.09.1 + 494; Bates et al., 2015; Christensen, 2018). Population was contrast coded as –0.5 (non-autistic) and 0.5 (autistic). Motivation was coded as –0.5 (protective) and 0.5 (beneficial). Orientation had three levels with the referent category coded as other orientation, and self and pareto orientations coded as comparison categories. When the final model included a significant orientation effect, the model was repeated with self as the referent category to compare self and pareto orientations. Experiences of lying as dependent measures were coded as 1–7. Reasons for telling the truth and emotions after telling the truth as dependent measures were coded as 0 or 1.

All models were built up sequentially, adding fixed effects individually and comparing each model with the previous best fitting model using log-likelihood ratio tests. Each analysis started with a baseline model containing by-participant, by-scenario and by-questionnaire-order intercepts with random slopes of motivation per participant and orientation × population slopes per scenario. This allowed us to account for variability across the different lying scenarios, variability across each participant’s responses and potential order effects. If models failed to converge, random effects were simplified until all models in the sequence successfully converged. Only final models are reported; please refer to Supplementary Materials for full details of model-building sequences. See Table 3 for each population’s probability of lying per scenario and Table 4 for descriptive statistics for all dependent measures.

**Table 3.** Probability that autistic (ASD) and non-autistic (NA) adults would lie in each situation in the lying scenario questionnaire.

Lie type	Scenario	% of sample who chose to lie	
		ASD	NA
Self-beneficial	Lost money	7.14	8.62
	Restaurant	14.30	8.62
Self-protective	Seminar	67.90	74.10
	Damaged book	8.93	6.90
Other beneficial	Surprise party	53.60	67.20
	New hat	62.50	62.10
Other protective	Truant friend	87.50	93.10
	Group work	55.40	70.70
Pareto beneficial	Pub quiz	12.50	20.70
	Booking table	3.57	15.50
Pareto protective	Sports game	28.60	46.60
	Lift breaks	53.60	75.90

**Table 4.** Descriptive statistics for autistic (ASD) and non-autistic (NA) adults’ likelihood of lying across scenarios and their emotional experience related to lying and truth-telling.

Dependent variable		ASD	NA
		*M* (*SD*)	*M* (*SD*)
Lie frequency (0 = True to 1 = Lie)		0.38 (0.49)	0.46 (0.50)
	Guilt	3.08 (1.86)	2.71 (1.63)
Experience of lying (1–7)	Believed	4.43 (1.51)	4.81 (1.37)
	Difficult	3.05 (1.69)	2.60 (1.53)
	Guilt	0.32 (0.47)	0.22 (0.41)
	Proud	0.10 (0.30)	0.11(0.32)
Emotions after telling the truth (0 = No to 1 = Yes)	Relieved	0.22 (0.41)	0.24 (0.43)
	Relaxed	0.19 (0.39)	0.24 (0.43)
	Happy	0.05 (0.22)	0.06 (0.23)

Participants were able to select multiple answers for emotions experienced after truth-telling.

#### Lie frequency

**Figure 2.**
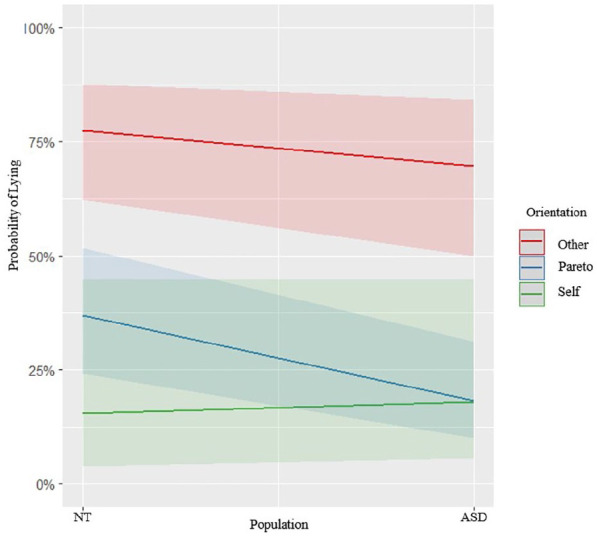
Visualisation of the population × orientation interaction detected for lie frequency, indicating differences in autistic and non-autistic adults’ responses depending on orientation.

Lie frequency was analysed via generalised linear mixed-effects models testing the effects of population, motivation and orientation. This analysis contained 1368 data points. The best fitting model included fixed effects of motivation (*z* = –4.02, *p* < 0.001) and orientation (other vs pareto, *z* = –4.30, *p* < 0.001, other vs self, *z* = –3.36, *p* < 0.001, self vs pareto, *z* = 0.80, *p* = 0.421; see Table 5). Participants across groups were more likely to lie when the motivation was protective compared to beneficial. Participants across groups were more likely to lie in other-oriented scenarios compared to pareto-oriented and self-oriented scenarios. There was also a significant population × orientation interaction (*z* = –2.75 *p* = 0.006; see Figure 2). This interaction was deconstructed by testing the effect of population at each level of orientation separately. The effect of population was significant for pareto-oriented scenarios (*z* = –3.69, *p* < 0.001), but not for self- (*z* = 0.36, *p* = 0.722) or other-oriented (*z* = –1.68, *p* = 0.094) scenarios. These findings indicate that autistic adults were less likely than non-autistic adults to lie in pareto-oriented scenarios, but not other- or self-oriented scenarios.

**Table 5.** Summaries of the fixed effects in the final generalised linear mixed-effects models (log odds) of lie frequency for the hypothetical lying scenarios.

	Fixed effects	Estimated coefficient	Standard error	*z*	Pr(>|*z*|)
Lie frequency	(Intercept)	1.03	0.38	2.76	0.006
	Population	–0.43	0.26	–1.66	0.097
	Orientation (Pareto vs Other)	–2.03	0.47	–4.30	< 0.001
	Orientation (Self vs Other)	–2.64	0.79	–3.36	< 0.001
	Orientation (Self vs Pareto)	0.60	0.75	0.80	0.421
	Motivation	–1.72	0.43	–4.02	< 0.001
	Population × Orientation (Pareto vs Other)	–0.53	0.34	–1.58	0.114
	Population × Orientation (Self vs Other)	–0.54	0.38	1.40	0.162
	Population × Orientation (Self vs Pareto)	–1.07	0.38	–2.75	0.006
		AIC	BIC	logLik	Deviance
		1402.7	1569.8	–669.4	1336

AIC: Akaike information criterion; BIC: Bayes information criterion.

#### Experiences of lying

Participants’ emotional experiences when lying were explored using cumulative link models testing the effects of population, motivation and orientation. Each analysis contained 574 data points.

##### Guilt

The final model included a significant fixed effect of population (*z* = 2.26, *p* = 0.024; see Table 6). Autistic participants reported that they would experience more guilt when lying compared to non-autistic adults.

**Table 6.** Summaries of the fixed effects in the final generalised linear mixed-effects models (log odds) related to the experience of lying (including guilt, difficulty and believability).

	Fixed effects	Estimated coefficient	Standard error	*z*	Pr(>|*z*|)
Guilt	Population	0.67	0.30	2.26	0.024
		AIC	Niter	logLik	
		1984.56	1792 (8888)	–975.28	
	Fixed effects	Estimated coefficient	Standard error	*z*	Pr(>|*z*|)
Believed	Population	–0.65	0.27	–2.38	0.017
	Orientation (self vs other)	0.49	0.45	1.07	0.287
	Orientation (self vs pareto)	–2.28	0.49	–4.62	< 0.001
	Orientation (pareto vs other)	–1.80	0.43	–4.21	< 0.001
		AIC	Niter	logLik	
		1890.17	1752 (5279)	–926.09	
	Fixed effects	Estimated coefficient	Standard error	*z*	Pr (>|*z*|)
Difficulty	Population	0.66	0.30	2.18	0.029
		AIC	Niter	logLik	
		1988.10	1752 (7013)	–977.05	

AIC: Akaike information criterion.

##### Believed

The final model included significant fixed effects of population (*z* = –2.38, *p* = 0.017) and orientation (pareto vs other: *z* = –4.21, *p* < 0.001; pareto vs self: *z* = –4.62, *p* < 0.001; self vs other: *z* = 1.07, *p* = 0.287). Autistic participants felt they would be significantly less likely to be believed than non-autistic participants. Across groups, participants felt they would be significantly less likely to be believed in pareto-oriented scenarios compared to other- and self-oriented scenarios.

##### Difficulty

The final model included a significant fixed effect of population (*z* = 2.18, *p* = 0.029). Autistic participants reported that lying would be significantly more difficult for them than non-autistic participants.

#### Emotions after telling the truth

Emotions after telling the truth were analysed via generalised linear mixed-effects models testing the effects of population, motivation and orientation. Each analysis contained 794 data points.

##### Guilt

The final model included significant fixed effects of motivation (*z* = –8.76, *p* < 0.001) and orientation (other vs self; *z* = –3.37, *p* < 0.001, self vs pareto; *z* = 4.12, *p* < 0.001, pareto vs other; *z* = –0.61, *p* = 0.534; see Table 7). Across groups, participants reported they would feel more guilt when telling the truth in scenarios with protective motivations than beneficial motivations, and in scenarios that were other- and pareto-oriented rather than self-oriented.

**Table 7.** Summaries of the fixed effects in the final generalised linear mixed-effects models (log odds) predicting emotions experienced by participants after telling the truth in the hypothetical scenarios.

	Fixed effects	Estimated coefficient	Standard error	*z*	Pr(>|*z*|)
Guilty	(Intercept)	–0.50	0.47	–1.05	0.294
	Orientation (Other vs Pareto)	–0.31	0.49	–0.61	0.534
	Orientation (Other vs Self)	–2.03	0.60	–3.37	< 0.001
	Orientation (Self vs Pareto)	1.72	0.42	4.12	< 0.001
	Motivation	–2.80	0.32	–8.76	< 0.001
		AIC	BIC	logLik	Deviance
		766.0	901.6	–354.0	765
	Fixed effects	Estimated coefficient	Standard error	*z*	Pr(>|*z*|)
Relaxed	(Intercept)	–1.73	0.15	–11.42	< 0.001
	Motivation	1.72	0.26	6.56	< 0.001
		AIC	BIC	logLik	Deviance
		805.20	931.40	–374.60	751.20
	Fixed effects	Estimated coefficient	Standard error	*z*	Pr(>|*z*|)
Relieved	(Intercept)	–1.47	0.15	–9.51	< 0.001
		AIC	BIC	logLik	Deviance
		817.20	938.80	–382.60	765.20
	Fixed effects	Estimated coefficient	Standard error	*z*	Pr(>|*z*|)
Proud	(Intercept)	–7.78	2.00	–3.90	< 0.001
	Motivation	11.67	3.98	2.95	0.003
		AIC	BIC	logLik	Deviance
		480.30	606.60	–213.20	426.30
	Fixed effects	Estimated coefficient	Standard error	–	Pr(>|*z*|)
Happy	(Intercept)	–12.54	1103.62	–0.01	0.991
	Motivation	18.17	2207.24	0.01	0.993
		AIC	BIC	logLik	Deviance
		329.70	456.00	–137.90	257.70

AIC: Akaike information criterion; BIC: Bayes information criterion.

##### Relaxed

The final model included a significant fixed effect of motivation (*z* = 6.56, *p* < 0.001). Across groups, participants reported they would feel more relaxed after telling the truth when the motivation to lie was beneficial compared to protective.

##### Relief

The final model included no fixed effects or interactions. Population, motivation and orientation had no significant effects on participants reporting that they would feel relieved after telling the truth.

##### Proud

The final model included a significant fixed effect of motivation (*z* = 2.95, *p* = 0.003). Across groups, participants reported they would feel significantly prouder after telling the truth when their motivation to lie was beneficial rather than protective.

##### Happy

The final model included a fixed effect of motivation, but this effect was non-significant (*z* = 0.01, *p* = 0.993). Therefore, although the addition of motivation as a fixed effect improved model fit, motivation did not significantly predict how happy participants would feel after telling the truth.

## Discussion

This study examined whether autistic and non-autistic adults differ in their lying frequency and emotional experiences when lying. Crucially, we investigated both general lying patterns (e.g. how often people self-report lying in day-to-day life) and inclination to lie across a range of scenarios presenting different motivations (beneficial, protective) and orientations (self, other, pareto). When asked about general lying behaviour, there were no differences in lie frequency detected between populations. When asked whether they would lie in various social scenarios, autistic and non-autistic adults did not significantly differ in their inclination to tell self- and other-oriented lies. However, autistic adults were significantly less likely to tell pareto-oriented lies than non-autistic adults. These results suggest that similarities in self-reported general lying behaviour may not reflect deceptive decision-making in specific social situations. We also discovered differences in the experience of lying between populations, with autistic adults anticipating feeling less confident that they would be believed, increased perceived difficulty and expecting to feel more guilt when choosing to deceive. Finally, when examining participants’ predicted emotions after truth telling, both non-autistic and autistic participants were significantly more likely to anticipate feeling more relaxed after telling the truth in hypothetical scenarios with beneficial motivations compared to protective motivations.

### Lie frequency and lie type

#### General lying behaviour

Due to the increased complexity of deception in adulthood (Walczyk & Fargerson, 2019) and differences associated with social experience and cognitive functioning (Baron-Cohen, 2008), we predicted that autistic adults may lie less frequently than non-autistic adults in specific social scenarios but display similar levels of general lying behaviour. Aligning with the studies by Van Tiel et al. (2021) and Bagnall et al. (2023), our analysis of general lying behaviour indeed suggests that autistic and non-autistic adults do not differ in their general lie frequency. Our findings also demonstrate that specific aspects of general lying behaviour, such as the mediums through which lies are told, do not significantly differ between autistic and non-autistic adults. These findings challenge the stereotypical assumption that autistic adults cannot (or do not) lie (Baron-Cohen, 2008; Maras et al., 2019). However, these general data are restricted by the absence of context. That is, conclusions drawn about autistic adults’ general deceptive decision-making may not accurately represent their behaviour in specific social situations.

#### Specific lying behaviour

When examining inclination to lie in specific contexts, autistic adults were less likely to tell pareto-oriented lies (lying for a group). This context-sensitive reduction in lie frequency may be driven by autistic adults’ reduced exposure to situations that motivate pareto-oriented lies. To tell a pareto-oriented lie, one must belong to a group and engage in social interactions. However, due to differences in social communication and interaction with peers from a young age (Locke et al., 2010), some autistic adults may develop fewer, or weaker, group relations and value more time alone or in smaller groups (Calder et al., 2013; Dean et al., 2014). Consequently, autistic adults may be on the periphery of social networks, reducing their experience and understanding of situations that motivate pareto-oriented lies. Furthermore, autistic adults may intentionally curate their social lives to avoid group interactions (Chevallier et al., 2012), avoiding situations that prompt pareto-oriented lying. Although autistic and non-autistic adults’ general lie-telling frequency may not differ, our scenario data show that these populations do significantly differ in their self-reported likelihood of engaging in social lies for the benefit or protection of groups.

Comparatively, autistic and non-autistic adults did not differ in their inclination to tell self- and other-oriented lies. One potential explanation for this similarity in inclination to lie is that many autistic adults’ report having at least one strong friendship (Bauminger et al., 2008). Having similar social experiences related to interacting with a friend may explain why autistic and non-autistic adults would be just as likely to lie to benefit/protect another person or themselves in specific social situations (e.g. in the New Hat/Damaged Book scenarios, see Supplementary Materials, p.3).

It is possible that autistic adults’ deceptive decision-making in a given social situation may be influenced by the diagnostic status of their communication partners. Many autistic adults report feeling more comfortable with other autistic adults, increasing the likelihood of autistic–autistic social experiences and friendships (Crompton et al., 2020; Sinclair, 2010). Some autistic adults are believed to share an autism identity, which K. Cooper et al. (2017) define as a type of social identity that fosters feelings of psychological connection to other autistic adults. This sense of connection can increase attachment between autistic adults and the ability to provide social support (K. Cooper et al., 2017). In this study, when asked whether they would lie to benefit/protect a friend, autistic participants may have envisioned an autistic friend who they share a connection with, potentially increasing their inclination to lie. However, when asked to envision lying in group situations that they may not have experienced with autistic peers (e.g. a pub quiz or sports team), autistic participants may have perceived hypothetical group members to be non-autistic. Such perceptions of group members’ diagnostic status may have reduced autistic participants’ inclinations to lie in pareto-oriented situations. Had autistic participants been informed that group members were autistic, this could have conceivably increased their inclination to lie for the group’s benefit/protection. However, these speculations require validation in future research investigating how autistic adults’ lie telling differs across varied scenarios involving autistic–autistic and autistic–non-autistic interactions.

### Emotional experience of deceptive decision-making

#### Experience of lying

In line with the study by Bagnall et al. (2023), our results indicate that autistic adults anticipate experiencing heightened levels of guilt when lying, increased difficulty and are more likely than non-autistic adults to assume their deception would be discovered. One reason for anticipating increased guilt may be that some autistic adults experience an increased moral objection to deception compared to non-autistic adults (Dempsey et al., 2020; Jaarsma et al., 2012). Autistic adults may find lying more difficult and be less confident in their deceptive abilities than non-autistic adults due to spending less time in social interactions (Chevallier et al., 2012), reducing their exposure to deception and opportunities to learn and practice deceptive behaviours (Talwar & Crossman, 2011). Autistic adults’ increased anticipated difficulty of producing lies, and lower confidence in their success, may also reflect that engaging in deception is more cognitively demanding for them than for non-autistic adults (Bagnall et al., 2022; Blackhurst et al., 2024).

Despite autistic adults evaluating their lie-telling ability more negatively than non-autistic adults, there were no significant differences in self-reported lie frequency between populations. However, for non-autistic adults, there is a positive relationship between lie-telling ability and lying frequency (Verigin et al., 2019). One potential explanation for why autistic adults may choose to lie, despite finding it difficult, is because they feel pressure to mask their autistic-identity and ‘fit in’ with non-autistic peers by speaking or acting a certain way in social situations. A recent study discovered that over 70% of autistic adults reported that they consistently camouflaged during social interactions (Cage & Troxell-Whitman, 2019). Moreover, self-reported levels of camouflaging are consistently higher in autistic females than males (Hull et al., 2017; Tubío-Fungueiriño et al., 2021), potentially due to differences in stigmatisation between genders (e.g. facing the male-dominated narrative regarding what autism ‘looks like’; Saxe, 2017). Consequently, during interactions with non-autistic adults, autistic adults may lie about their hobbies and preferences or purposefully change or hide aspects of their personality and behaviour to appear more socially compatible (Cook et al., 2022). In such instances, lying would represent a protective strategy to avoid discrimination, potentially outweighing the negative emotions experienced during the lie itself (Blackhurst et al., 2024; Perry et al., 2022). Future research is required to explore the relationship between social camouflaging and everyday deception, providing autistic adults with opportunities to explain when and why they engage in deceit.

#### Experience of telling the truth

Although population did not influence participants’ expected emotions when truth-telling, both autistic and non-autistic adults anticipated feeling more relaxed if they were to tell the truth in scenarios with beneficial (rather than protective) motivations. This finding may reflect the awareness of both populations that telling the truth in beneficial scenarios may have fewer negative consequences than in protective scenarios (i.e. they/another may not receive a gain, but more importantly they would not experience a loss; see Schmidt & Traub, 2002), eliciting fewer concerns about the possible outcomes of their honesty. In addition, both populations anticipated feeling more guilt when telling the truth in scenarios with protective (rather than beneficial) motivations, perhaps indicating that both autistic and non-autistic adults are aware that their honesty may have a negative impact on themselves and/or others. Together, these results suggest that both autistic and non-autistic adults similarly recognise the potential benefits and/or consequences of choosing not to lie across social contexts with differing social motivations.

### Limitations

This study has several limitations. First, our use of scenario vignettes did not directly expose participants to pressures often present during naturalistic social interactions involving deception. As such, our data may not fully capture the true difficulty of deceptive decision-making for autistic adults. However, utilising vignettes allowed us to manipulate social context and reflect naturalistic variability by presenting a typology of everyday lies that vary in difficulty, level of acceptability, familiarity and severity. Although we controlled for scenario by using a random effect in the model, in line with our pre-registration, we did not ask participants to rate their subjective perceptions of each scenario. While it is possible that autistic adults’ reduction in hypothetical pareto-oriented lie frequency may be attributable to differences in their subjective perceptions of these scenarios, this account is not incompatible with our theoretical explanation regarding differences in social experience. That is, autistic adults may be less familiar with, and differ in their perceptions of, situations that elicit pareto-oriented lies because of their social experiences. It is also important to note that, within their samples, both autistic and non-autistic adults are likely to have varied substantially in terms of familiarity with the kinds of scenarios presented for each category of lie. Despite these unmeasured subjective factors, our data demonstrate clear similarities and differences in autistic and non-autistic adults’ lie-telling behaviour across varied and nuanced situations in which people may choose to deceive. Nevertheless, to advance understanding of how participants’ subjective experiences and perceptions influence their lie-telling decisions, future research may benefit from directly investigating the impact of perceived difficulty, level of acceptability, familiarity and severity on autistic adults’ lie frequency across varied social contexts.

Second, due to autism stigma (autistic people often experience ignorance and discrimination directed towards them; Turnock et al., 2022), some autistic adults report that their social cognitive ability is poorer than it truly is (DeBrabander et al., 2021). Therefore, inaccurate self-assessments of lie-telling ability stemming from the internalisation of stereotypical beliefs about autism (Bagnall et al., 2023; Han et al., 2022) may have inflated the observed between-population differences reported in lying experiences. To address this, future research may benefit from investigating how autistic adults’ experiences of stigma and their perceptions of non-autistic stereotypes influence self-assessments of lie-telling ability.

Finally, it is important to reflect on the extent to which our observed results may generalise to the broader autistic population. Our sample of autistic adults was predominantly female and consisted of individuals with high intellectual ability and verbal mental age (university students). As such, males and individuals with linguistic and intellectual difficulties were not proportionately represented in this study. Our sample may more accurately reflect the ‘female autism phenotype’ characterised by increased visual skills, higher IQ scores and sophisticated social camouflaging (Lai et al., 2015). Consequently, different patterns of results may be observed across samples with varying gender and cognitive profiles, and we recommend that future research explores these individual differences across the autistic population. Numerous reports have highlighted how individuals with intellectual disabilities may be at a higher risk of falsely confessing to crimes during police interviews (e.g. lying about committing a crime; Gudjonsson, 2018; Lloyd, 2017; Schatz, 2018). As such, there is a pressing need to investigate when and why autistic adults may lie (including those with lower intellectual abilities), particularly in forensic situations.

### Conclusion

This study advances theoretical knowledge of deception in autism as it is the first to employ a typology of lies to identify how autistic adults’ deceptive decision-making differs across social contexts. We discovered that autistic and non-autistic adults do not differ in their general lying behaviour, including frequency of lie-telling, who they lie to or the mediums through which they choose to lie. These findings contradict the common stereotypical assumption that autistic people cannot, or do not, lie. When examining context-specific deception, we found that autistic and non-autistic adults did not differ in their inclination to tell self- and other-oriented lies. However, autistic adults were significantly less likely to tell pareto-oriented lies. Pareto-oriented lies may be crucial to the maintenance of social relationships, as being unwilling to engage in pareto-oriented deception may have negative consequences for other group members. If autistic adults are less inclined to lie to benefit or protect social partners in a group, this may increase strain on their social bonds and potentially contribute to relationship breakdowns due to losing the trust of other group members. Furthermore, this study highlights explicit differences in the experience of lying between populations. Autistic adults reported that they would find lying more difficult and appear less confident in their ability to lie undetected. However, whether this self-assessment stems from genuine differences in lie-telling ability or internalisation of stereotypes requires further investigation. To conclude, this research highlights the risk of drawing potentially inaccurate conclusions about autistic behaviour from general measures of lying by demonstrating how autistic adults’ deceptive decision-making differs across social contexts.

## Supplemental Material

sj-docx-1-aut-10.1177_13623613251315892 – Supplemental material for Exploring lie frequency and emotional experiences of deceptive decision-making in autistic adults Supplemental material, sj-docx-1-aut-10.1177_13623613251315892 for Exploring lie frequency and emotional experiences of deceptive decision-making in autistic adults by Tiegan Blackhurst, Lara Warmelink, Amanda Roestorf and Calum Hartley in Autism
