# Supplementary material for: Exploring lie frequency and emotional experiences of deceptive decision-making in autistic adults
Source: Autism. 2025 Mar 3;29(6):1612–26. doi: 10.1177/13623613251315892 (PMC12089678; doi:10.1177/13623613251315892)
Supplement: sj-docx-1-aut-10.1177_13623613251315892 – Supplemental material for Exploring lie frequency and emotional experiences of deceptive decision-making in autistic adults [file sj-docx-1-aut-10.1177_13623613251315892.docx]

Supplementary materials outlining full model building sequences for the results in the manuscript, full analysis of justification for telling the truth variables, the lying questionnaires used during this study, and examples of all lie-scenarios.

***General Lying Questionnaire:***

In general how often do you lie?

1. Never
2. Less than once a month
3. 2-3 times a month
4. Once a week
5. 2-3 times a week
6. Once a day
7. 2-5 times a day
8. 5-10 times a day
9. 10+ times a day

In general, when you lie how guilty do you feel?

0 (Not at all,), 1, 2 (Not very Guilty), 3, 4 (Quite Guilty), 5, 6

In general, when you lie how often are you believed?

0 (Never), 1, 2, 3, 4, 5, 6 (Always)

In general, how difficult do you find it to lie to people?

0, (Not at all), 1, 2, (Not Very), 3, 4, (Quite), 5, 6 (Extremely)

*
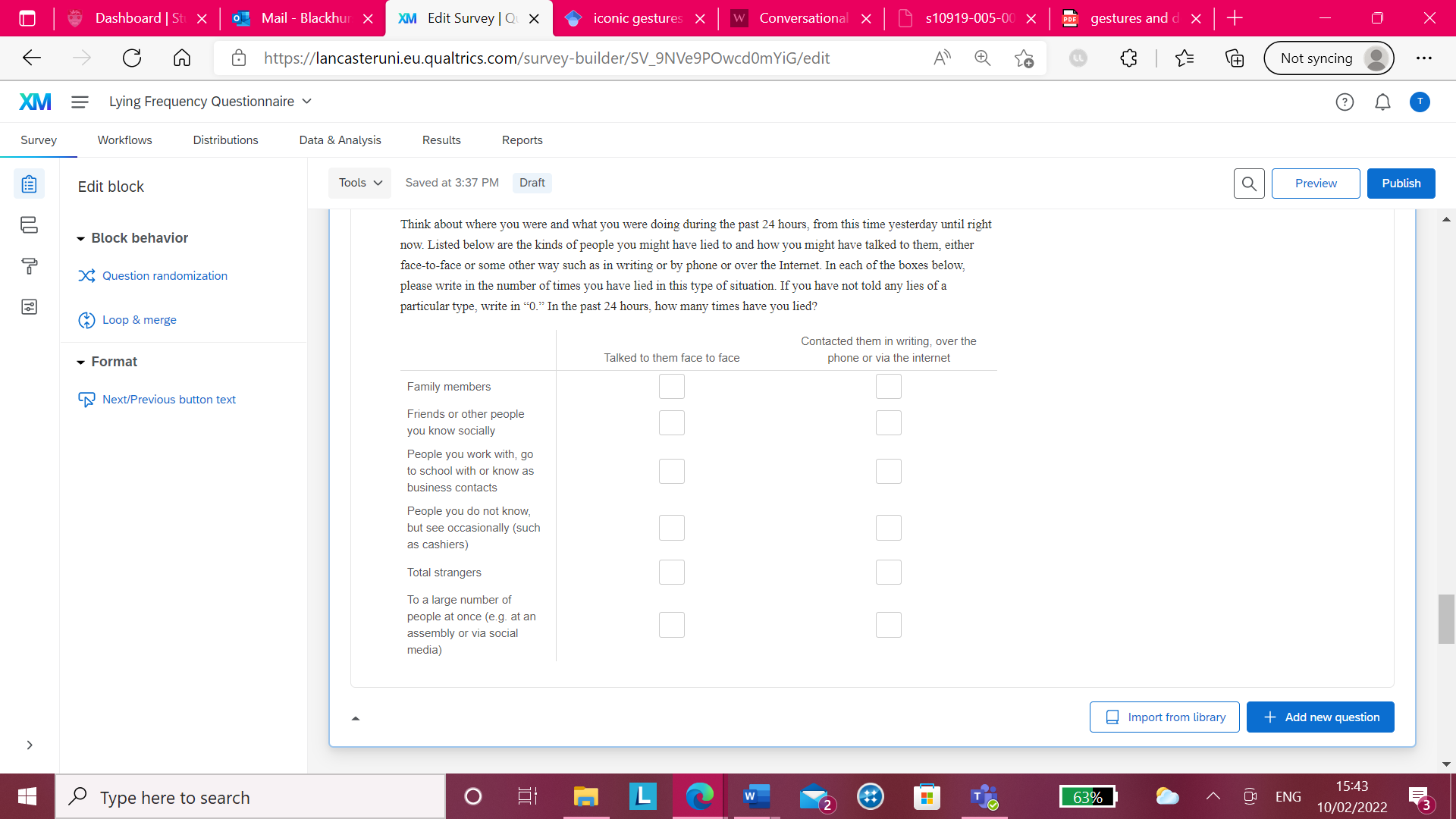
****Serota Grid:***

**Table 1.**

*Lie Scenarios.*

| Name of Scenario | Orientation | Motivation | Scenario |
| --- | --- | --- | --- |
| Damaged Book | Self | Protective | Imagine your best friend lends you an item that they were very fond of (e.g. a favourite book, or an item of clothing). You accidentally damage the item. You have no choice but to tell your friend that the item is broken. However, you realise you could blame the accident on someone else (e.g. an acquaintance you are not particularly close to). Would you lie and say the accident was somebody else's fault ? |
| Seminar | Self | Protective | Imagine you have gone to a psychology seminar, for which you should have read a document. You had previously downloaded the document and it opened correctly, but you didn’t actually read it. You know your seminar tutor will be disappointed and angry that you haven’t prepared for your class. In the class, the tutor says that they heard that some people had received a corrupted version of the document and were not able to read the text. They ask whether your document was also corrupted.  Would you lie and say that your document was also corrupted? |
| Lost Money | Self | Beneficial | Imagine you are sat in a café finishing off a university assignment. The waitress finds £10 on the floor near your table. You have not lost any money and know that this money is not yours, however you are the only customer left in the café. The waitress comes over and asks whether you dropped the £10?  Would you lie and say the money was yours? |
| Restaurant | Self | Beneficial | Imagine you are in a restaurant with a large group of acquaintances. You had to pre-order your food days in advance.  You ordered something you normally like, but on this occasion you don't feel like eating it. The waitress comes out with several plates of food that you would really like to eat, but they are not what you pre-ordered. Nobody has kept clear track of who had ordered what.  Would you lie and say the food you want right now is what you ordered? |
| Group Work | Other | Protective | Imagine you are chatting to a fellow student, who mentions that she has problems finding a partner for some group work that your have to complete for your module. They ask whether you know why other students won’t work with them. You know it is because your fellow student is considered to be bad at group work, because they are often ill. You know that telling your fellow student this would hurt their feelings.  Would you lie and either say you don't know or give a different reason? |
| Truant Friend | Other | Protective | Imagine you and your friend always travel into university together. One day there is a large social event near your university and your friend has told you that they are going to tell the university administration that they are ill, so he/she can miss a compulsory seminar and go to the event instead. At university, your seminar tutor suspects that your friend is not really sick, but is skipping the seminar. Your seminar tutor asks you whether you know where your friend is.  Would you lie and say your friend is sick? |
| Surprise Party | Other | Beneficial | Imagine you and your friends are organising a surprise party for your best friend's birthday, which you all know he/she will love. To arrange the party, you have had to exclude your best friend from conversations and meetings.  Your best friend is starting to feel left out. A couple of days before the surprise party, your best friend tells you that they are hurt by this behaviour and ask you whether you know what is going on?  Would you lie and keep the surprise party from your friend? |
| New Hat | Other | Beneficial | Imagine your best friend comes over to see you and they are wearing a new hat. Your best friend tells you that they bought the hat yesterday and that they love it. Your friend then asks whether you like their hat. You take a closer look at the hat and decide that you hate it but you know that this would upset your friend if you were to tell them.  Would you lie and say you loved the hat? |
| Sports Game | Pareto | Protective | Imagine you are a member of a sports team. You have a big match on Saturday but the whole team, including yourself, decided to go out on Friday night. You and the team only got home at 5am on Saturday morning and you all turned up to the match visibly tired. You know that your coach does not like the team going out the day before a match and he would be angry if he knew you had all stayed out late. Your coach can tell something is wrong with the team and asks if you all went out the night before.  Would you lie and say you and the team did not go out? |
| Lift Breaks | Pareto | Protective | Imagine you and 7 of your friends are about to step into a lift until you see a sign that says the capacity of the lift is 5. You disregard this and all enter the lift anyway thinking it will be okay but the lift stops breaks down half way and the emergency services are called to rescue you. This costs the emergency services precious time and resources and when they rescue you and your friends from the lift, they see that you have exceeded the lifts capacity and this is why it broke down. They ask whether you saw the sign with the capacity of the lift?  Would you lie and say none of you saw the sign with the lifts capacity on? |
| Booking Table | Pareto | Beneficial | Imagine you and your friends are going out for a meal. When you get to the restaurant you all realise that nobody has booked a table and the restaurant looks full. You all really wanted to try this restaurant as you had heard the food was very good. When speaking with the front-of-house staff you realise that you could say you had made a booking for you and your friends and it must be a mistake on the system if there is no record of it.  Would you lie and say you made the booking for you and your friends? |
| Pub Quiz | Pareto | Beneficial | Imagine you are competing in a pub quiz with your friends. The prize is £100. To win the money you and your team had to get 30 points; your team have scored 29. You know that you could tell the quiz master that you and your team got 30 points and you would win the prize money and could split it between you.  Would you lie and say you had enough points to win the money? |

***Questions following the hypothetical social scenarios:***

**If participants stated they would lie:**

How guilty would you feel if you were to lie in the scenario as described?

0 (Not at all), 1, 2 (Not very Guilty), 3, 4 (Quite Guilty), 5, 6 (Extremely)

How confident are you that your lie would be believed?

1 (I’m sure it wouldn’t be), 2, 3, 4 (I don’t know), 5, 6, 7 (I’m sure it would be)

How difficult do you think it would be to lie?

0 (Not at all), 1, 2, 3, 4, 5, 6 (Extremely)

**If participants stated they would tell the truth:**

Why would you not lie in the scenario as described? (Multiple selection allowed)

- I don’t think I’d be believed
- It would make me feel bad
- It would be morally wrong
- Other (Please state)

How would you feel after telling the truth? (Multiple selection allowed)

- Proud
- Relaxed
- Guilty
- Happy
- Relieved
- Other

***Reasons for Telling the Truth Results***

Reasons for telling the truth were analysed via generalised linear mixed-effects models testing the effect of population, motivation and orientation. Each analysis contained 800 data points.

*Fear of Not Being Believed:*

The final model included significant fixed effects of motivation (z = -3.19, *p* = .029) and orientation (self vs. other; z = -1.98, *p* = .005, pareto vs. self; z = 2.13, *p* <.001; see Table 6). There was no significant effect for orientation pareto vs. other (z = 0.25, *p* = .806). Across groups, participants were significantly more likely to select fear of not being believed as a reason for telling the truth when motivation was protective compared to beneficial, and in other/ pareto-oriented scenarios compared to self-oriented scenarios.

*Morally Wrong*:

The final model included a significant fixed effect of population (z = 2.23, *p* = .026) and orientation (other vs. self; z = 3.24 *p* = .001; see Table 6). Autistic adults were significantly more likely than neurotypical adults to tell the truth because lying would be morally wrong. Across groups, participants were significantly more likely to cite morality as the reason for telling the truth in self-oriented scenarios compared to other-oriented scenarios.

We also detected a significant motivation x orientation interaction (other vs. pareto * motivation). This interaction was deconstructed by testing the effect of motivation at each level of orientation separately. There was a significant main effect discovered between motivation and pareto orientations (*z* = 2.44, *p* = .015) and motivation and other orientations (*z* = -1.97, *p* = .049). Across groups, participants were significantly more likely to cite morality as the reason for telling the truth in other-oriented scenarios when motivation was protective and in pareto-oriented scenarios when motivation was beneficial.

**Table 2**

|  | **Fixed effects** | **Estimated coefficient** | **Std. error** | **z** | **Pr(>\|z\|)** |
| --- | --- | --- | --- | --- | --- |
| Not Believed | (Intercept) | -0.73 | 0.52 | -1.40 | .160 |
|  | Motivation | -1.13 | 0.51 | -3.19 | .029 |
|  | Orientation (other vs pareto) | 0.15 | 0.62 | 0.25 | .806 |
|  | Orientation (other vs self) | -1.98 | 0.70 | -2.82 | .005 |
|  | Orientation (self vs pareto) | 2.13 | 0.62 | 3.44 | <.001* |
|  |  | **AIC** | **BIC** | **logLik** | **deviance** |
|  |  | 732.70 | 859.30 | -332.80 | 665.70 |
|  | **Fixed effects** | **Estimated coefficient** | **Std. error** | **z** | **Pr(>\|z\|)** |
| Morally Wrong | Intercept | -1.79 | 0.34 | -5.29 | <.001 |
|  | Population | 0.55 | 0.25 | 2.23 | .026 |
|  | Orientation (other vs pareto) | 0.99 | 0.65 | 1.52 | .129 |
|  | Orientation (other vs self) | 1.84 | 0.57 | 3.24 | .001 |
|  | Orientation (self vs pareto) | -0.80 | 0.73 | -1.09 | .276 |
|  |  | **AIC** | **BIC** | **logLik** | **deviance** |
|  |  | 987.7 | 1123.4 | -464.9 | 765 |
|  | **Fixed effect** | **Estimated coefficient** | **Std. error** | **z** | **Pr(>\|z\|)** |
| Feel Bad | (Intercept) | -0.96 | 0.17 | -5.58 | <.001* |
|  |  | **AIC** | **BIC** | **logLik** | **deviance** |
|  |  | 1033.4 | 1155.0 | -490.7 | 768 |

*Summaries of the fixed effects in the final generalised linear mixed effects models (log odds) of the reasons why participants told the truth in the hypothetical scenarios*

**Model Building Sequences for the Results Reported in the Main Text**

The hypothetical lying scenarios data were analysed via mixed effects linear and cumulative link models using glmer and clmm functions from the lme4 and ordinal package in R (Version /2023.09.1+494; Bates et al., 2015; Christensen, 2018). Population was contrast coded as -0.5 (neurotypical) and 0.5 (autistic). Motivation was coded as -0.5 (protective) and 0.5 (beneficial). Orientation had three levels with the referent category coded as other, and self and pareto coded as comparison categories. The analysis was run with self as the referent category if there was a significant effect of orientation to examine self vs. pareto effects.

All models were built up sequentially, adding fixed effects individually and comparing each model with the previous best fitting model using log-like-likelihood tests. Each analysis started with a baseline model containing by-participant, by-scenario, and by-questionnaire-order intercepts with a random slope of motivation per participant and orientation x population slopes per scenario. If some models in the sequence failed to converge, random effects were simplified until all models in the sequence successfully converged (the final random effects structure for each variable are provided below).

**Lie Frequency:**

We began with a baseline model containing by-participant, by-scenario, and by-questionnaire-order intercepts with a random slope of motivation per participant and orientation x population slopes per scenario. Fixed effects of Population (Model 2), Orientation (Model 3) and Motivation (Model 4) were entered individually. The addition of Motivation (χ^2^ = 5.06, *p* = .024) and population (χ^2 =^ 4.18, *p* = .041) yielded a significant improvement in fit when compared with the baseline model. The addition and Orientation (*p*= .065) did not significantly improve fit. Model 5 included fixed effects of Population + Orientation but did not significantly differ from Model 2 (*p* =.112) or 4 (*p* =.072). Model 6 included fixed effects of Population + Motivation and yielded a significant improvement in fit compared to Model 2 (χ^2^ = 5.26, *p* = .021) and Model 4 (χ^2^ = 4.38, *p* = .036). Model 7 included fixed effects of Motivation + Orientation and yielded a significant improvement in fit when compared with Model 2 (χ^2^ = 9.16, *p* =.010). and Model 4 (χ^2^ = 8.28, *p* =.015). Model 8 included fixed effects of Population + Motivation + Orientation and yielded a significant improvement in fit when compared with Model 6 (χ^2^ = 3.90, *p* =.048) but not Model 7 (*p* =.052). Model 9 included a fixed effect of Motivation and a two-way interaction between Population x Orientation and yielded a significant improvement in fit when compared with Model 6 (χ^2^ = 14.75, *p* =.005) and Model 7 (χ^2^ = 10.85 *p* = .013). Model 10 included a fixed effect of Population with a two-way interaction between Orientation x Motivation and yielded a significant improvement in fit when compared to Model 7 (χ^2^ = 9.17, *p* =.010) but not Model 6 (*p* = .104). Model 11 included a fixed effect of Orientation with a two-way interaction of Population x Motivation but did not significantly improve fit when compared with Model 6 (*p* = .077) or Model 7 (*p* = .209). Finally, the three-way interaction (Population x Motivation x Orientation) was entered (Model 12); doing so did not significantly improve fit in comparison to Model 6 (*p* = .051), Model 7 (*p* = .114) or Model 9 (*p* = .837). Thus, Model 9, containing a fixed effect of motivation and a two-way interaction of Population x Orientation, provided the best fit to the observed data.

***General Guilt When Lying:*** We initially began with a baseline model containing by-participant, by-scenario, and by-questionnaire-order intercepts with a random slope of motivation per participant and orientation x population slopes per scenario. However, this model failed to converge. We reduced the complexity of the random effects by removing the interaction between the random slope of Orientation x Population per scenario and replacing this with Orientation + Population. However, not all models in this sequence would converge. Therefore, guided by our predictions concerning the effects of population and orientation, we simplified the random effect structure even further by removing Motivation from the slope structure.

We began with a baseline model containing by-participant, by-scenario, and by-questionnaire-order intercepts with a random slope of orientation per participant and per scenario. Fixed effects of Population (Model 2), Orientation (Model 3) and Motivation (Model 4) were entered individually. The addition of Population (χ^2^ = 5.08, *p* = .024) yielded a significant improvement in fit when compared with the baseline model. The addition of Motivation (*p* = .559) and Orientation (*p*= .371) did not significantly improve fit when compared with baseline. Model 5 included fixed effects of Population + Orientation but did not significantly differ from Model 2 (*p* =.443). Model 6 included fixed effects of Population + Motivation but did not differ significantly from Model 2 (*p*= .560). Model 7 included fixed effects of Motivation + Orientation but did not differ significantly from Model 2 (*p* = 1.00). Model 8 included fixed effects of Population + Motivation + Orientation but did not significantly improve fit when compared with Model 2 (*p* =.558). Model 9 included a fixed effect of Motivation and a two-way interaction between Population x Orientation but did not significantly improve fit when compared with Model 2 (*p* = .679). Model 10 included a fixed effect of Population with a two-way interaction between Orientation x Motivation but did not significantly improve fit when compared with Model 2 (*p* = .340). Model 11 included a fixed effect of Orientation with a two-way interaction of Population x Motivation but did not significantly improve fit when compared with Model 2 (*p* = .438). Finally, the three-way interaction (Population x Motivation x Orientation) was entered (Model 12); doing so did not significantly improve fit in comparison to Model 2 (*p* =.201). Thus, Model 2, containing a fixed effect of Population, provided the best fit to the observed data.

***General Believability when Lying***

We initially began with a baseline model containing by-participant, by-scenario, and by-questionnaire-order intercepts with a random slope of motivation per participant and orientation x population slopes per scenario. However, this model failed to converge. We reduced the complexity of the random effects by removing the interaction between the random slope of Orientation x Population per scenario and replacing this with Orientation + Population. However, not all models in this sequence would converge. Therefore, guided by our predictions concerning the effects of population and orientation, we simplified the random effect structure even further by removing Motivation from the slope structure.

We began with a baseline model containing by-participant, by-scenario, and by-questionnaire-order intercepts with a random slope of orientation per participant and per scenario. Fixed effects of Population (Model 2), Orientation (Model 3) and Motivation (Model 4) were entered individually. The addition of Population (χ^2^ = 6.77, *p* = .009) and Orientation (χ^2^ = 15.40, *p* = <.001) yielded a significant improvement in fit when compared with the baseline model. The addition of Motivation (*p* = .322) did not significantly improve fit when compared with the baseline model. Model 5 included fixed effects of Population + Orientation and yielded a significant improvement in fit when compared with Model 2 (χ^2^ = 13.87, *p* = .001) and Model 3 (χ^2^ = 5.24, *p* = .022). Model 6 included fixed effects of Population + Motivation but did not differ significantly from Model 2 (*p* =.287) or Model 3 (*p* = 1.00). Model 7 included fixed effects of Motivation + Orientation and significantly improved fit when compared to Model 2 (χ^2^ = 10.37, *p* = .005) but not Model 3 (*p* =.137). Model 8 included fixed effects of Population + Motivation + Orientation but did not significantly improve fit when compared with Model 5 (*p* =.074). Model 9 included a fixed effect of Motivation and a two-way interaction between Population x Orientation but did not significantly improve fit when compared with Model 5 (*p* = .079). Model 10 included a fixed effect of Population with a two-way interaction between Orientation x Motivation but did not significantly improve fit when compared with Model 5 (*p* = .090). Model 11 included a fixed effect of Orientation with a two-way interaction of Population x Motivation but did not significantly improve fit when compared with Model 5 (*p* = .214). Finally, the three-way interaction (Population x Motivation x Orientation) was entered (Model 12); doing so did not significantly improve fit in comparison to Model 5 (*p* =.243). Thus, Model 5, containing a fixed effect of Population and Motivation, provided the best fit to the observed data.

***General Difficulty with Lying:***

We initially began with a baseline model containing by-participant, by-scenario, and by-questionnaire-order intercepts with a random slope of motivation per participant and orientation x population slopes per scenario. However, this model failed to converge. We reduced the complexity of the random effects by removing the interaction between the random slope of Orientation x Population per scenario and replacing this with Orientation + Population.

So, we began with a baseline model containing by-participant, by-scenario, and by-questionnaire-order intercepts with a random slope of motivation per participant and orientation + population slopes per scenario. Fixed effects of Population (Model 2), Orientation (Model 3) and Motivation (Model 4) were entered individually. The addition of Population (χ^2^ = 4.02, *p* = .035) yielded a significant improvement in fit when compared with the baseline model. The addition of Motivation (*p* = .873) and Orientation (*p*= .367) did not significantly improve fit when compared with the baseline model. Model 5 included fixed effects of Population + Orientation but did not significantly improve fit when compared with Model 2 (*p* =.307). Model 6 included fixed effects of Population + Motivation but did not differ significantly from Model 2 (*p*= .801). Model 7 included fixed effects of Motivation + Orientation but did not differ significantly from Model 2 (*p* = 1.00). Model 8 included fixed effects of Population + Motivation + Orientation but did not significantly improve fit when compared with Model 2 (*p* =.492). Model 9 included a fixed effect of Motivation and a two-way interaction between Population x Orientation but did not significantly improve fit when compared with Model 2 (*p* = .449). Model 10 included a fixed effect of Population with a two-way interaction between Orientation x Motivation but did not significantly improve fit when compared with Model 2 (*p* = .514). Model 11 included a fixed effect of Orientation with a two-way interaction of Population x Motivation but did not significantly improve fit when compared with Model 2 (*p* = .659). Finally, the three-way interaction (Population x Motivation x Orientation) was entered (Model 12); doing so did not significantly improve fit in comparison to Model 2 (*p* =.670). Thus, Model 2, containing a fixed effect of Population, provided the best fit to the observed data.

***Emotions are Telling the Truth***

***Guilt:***

We began with a baseline model containing by-participant, by-scenario, and by-questionnaire-order intercepts with a random slope of motivation per participant and orientation x population slopes per scenario. Fixed effects of Population (Model 2), Orientation (Model 3) and Motivation (Model 4) were entered individually. The addition of Population (χ^2^ = 5.33 *p* = .021) and Motivation (χ^2^ = 14.82, *p* <.001) yielded a significant improvement in fit when entered individually and compared to baseline. The addition of Orientation did not yield a significant improvement in fit (*p* = .071). Model 5 included fixed effects of Population + Orientation but did not significantly improve fit when compared to Model 2 (*p* = .339) or Model 4 (*p* = 1.00). Model 6 included fixed effects of Population + Motivation and yielded a significant improvement in fit when compared to Model 2 (χ^2^ = 14.10, *p* <.001) and Model 4 (χ^2^ = 4.62, *p* = .032). Model 7 included fixed effects of Motivation + Orientation and yielded a significant improvement in fit when compared to Model 2 (χ^2^ = 8.50, *p* = .014) and Model 4 (χ^2^ = 17.98, *p* <.001). Model 8 included fixed effects of Population + Motivation + Orientation but did not significantly improve fit when compared with Model 6 (*p* = .054) or Model 7 (*p* = .161). Model 9 included a fixed effect of Motivation and a two-way interaction between Population x Orientation but did not significantly improve fit when compared with Model 6 (*p* = .345) or Model 7 (*p* = .126). Model 10 included a fixed effect of Population with a two-way interaction between Orientation x Motivation and yielded a significant improve fit when compared with Model 6 (χ^2^ = 10.28, *p* = .036) but not when compared to Model 7 (*p* = .094). Model 11 included a fixed effect of Orientation with a two-way interaction of Population x Motivation but did not significantly improve fit when compared Model 6 (*p* = .107) or Model 7 (*p* = .330). Finally, the three-way interaction (Population x Motivation x Orientation) was entered (Model 12); doing so did not significantly improve fit in comparison to Model 6 (*p* = .171) or Model 7 (*p* = .346). Thus, to find the best fitting model to represent the data, the AIC & BIC values were compared for Model 6 (AIC= 767.84, BIC = 898.80 and Model 7 (AIC= 765.96, BIC = 901.60). As Model 7 has the lowest AIC, Model 7 containing a fixed effect of Motivation and Orientation provided the best fit to the observed data.

***Relaxed:***

We began with a baseline model containing by-participant, by-scenario, and by-questionnaire-order intercepts with a random slope of motivation per participant and orientation x population slopes per scenario. Fixed effects of Population (Model 2), Orientation (Model 3) and Motivation (Model 4) were entered individually. The addition of Population (*p* = .361) and Orientation (*p*= .252) did not significantly improve fit when compared with the baseline model. However, the addition of Motivation yielded a significant improvement in fit when compared with the baseline model (χ^2^= 15.07, *p* <.001). Model 5 included fixed effects of Population + Orientation but did not significantly improve fit when compared with Model 4 (*p* = 1.00). Model 6 included fixed effects of Population + Motivation but did not significantly improve fit when compared with Model 4 (*p*= .181). Model 7 included fixed effects of Motivation + Orientation and did not significantly improve fit when compared with Model 4 (*p* =.458). Model 8 included fixed effects of Population + Motivation + Orientation but did not significantly improve fit when compared with Model 4 (*p* =.456). Model 9 included a fixed effect of Motivation and a two-way interaction between Population x Orientation and did not significantly improve in fit when compared with Model 4 (*p* = .386). Model 10 included a fixed effect of Population with a two-way interaction between Orientation x Motivation which did not significantly improve in fit when compared with Model 4 (*p* = .249). Model 11 included a fixed effect of Orientation with a two-way interaction of Population x Motivation but did not significantly improve fit when compared with Model 4 (*p* = .629). Finally, the three-way interaction (Population x Motivation x Orientation) was entered (Model 12); doing so did not significantly improve fit in comparison to Model 4 (*p* =.446). Thus, Model 4, containing a fixed effect Motivation alone provided the best fit to the observed data.

***Relieved:***

We began with a baseline model containing by-participant, by-scenario, and by-questionnaire-order intercepts with a random slope of motivation per participant and orientation x population slopes per scenario. Fixed effects of Population (Model 2), Orientation (Model 3) and Motivation (Model 4) were entered individually. The addition of Population (*p* = .823) or Orientation (*p*= .122) or Motivation (*p* = .675) did not significantly improve fit when compared with the baseline model. Model 5 included fixed effects of Population + Orientation but did not significantly improve fit when compared with the baseline (*p* =.196). Model 6 included fixed effects of Population + Motivation but did not significantly improve fit when compared with the baseline (*p* =.894). Model 7 included fixed effects of Motivation + Orientation and did not significantly improve fit when compared with the baseline (*p* = .168). Model 8 included fixed effects of Population + Motivation + Orientation but did not significantly improve fit when compared with baseline (*p* =.222). Model 9 included a fixed effect of Motivation and a two-way interaction between Population x Orientation and did not significantly improve in fit when compared with baseline (*p* = .436). Model 10 included a fixed effect of Population with a two-way interaction between Orientation x Motivation which did not significantly improve in fit when compared with baseline (*p* = .185). Model 11 included a fixed effect of Orientation with a two-way interaction of Population x Motivation and did not yield a significant improve fit when compared with baseline (*p* = .073). Finally, the three-way interaction (Population x Motivation x Orientation) was entered (Model 12); doing so did not significantly improve fit in comparison to Model 11 (*p* =.052). Thus, the baseline model including no fixed effects or interactions provided the best fit to the observed data.

***Proud:***

We began with a baseline model containing by-participant, by-scenario, and by-questionnaire-order intercepts with a random slope of motivation per participant and orientation x population slopes per scenario. Fixed effects of Population (Model 2), Orientation (Model 3) and Motivation (Model 4) were entered individually. The addition of Population (*p* = .133) and Orientation (*p*= .594) did not significantly improve fit when compared with the baseline model. However, the addition of Motivation yielded a significant improvement in fit when compared with the baseline model (χ^2^= 31.75, *p* <.001). Model 5 included fixed effects of Population + Orientation but did not significantly improve fit when compared with Model 4 (*p* = 1.00). Model 6 included fixed effects of Population + Motivation but did not significantly improve fit when compared with Model 4 (*p*= .226). Model 7 included fixed effects of Motivation + Orientation and did not significantly improve fit when compared with Model 4 (*p* =.424). Model 8 included fixed effects of Population + Motivation + Orientation but did not significantly improve fit when compared with Model 4 (*p* =.395). Model 9 included a fixed effect of Motivation and a two-way interaction between Population x Orientation and did not significantly improve in fit when compared with Model 4 (*p* = .559). Model 10 included a fixed effect of Population with a two-way interaction between Orientation x Motivation which did not significantly improve in fit when compared with Model 4 (*p* = .258). Model 11 included a fixed effect of Orientation with a two-way interaction of Population x Motivation but did not significantly improve fit when compared with Model 4 (*p* = .512. Finally, the three-way interaction (Population x Motivation x Orientation) was entered (Model 12); doing so did not significantly improve fit in comparison to Model 4 (*p* =717). Thus, Model 4, containing a fixed effect Motivation alone provided the best fit to the observed data.

***Happy:***

We began with a baseline model containing by-participant, by-scenario, and by-questionnaire-order intercepts with a random slope of motivation per participant and orientation x population slopes per scenario. Fixed effects of Population (Model 2), Orientation (Model 3) and Motivation (Model 4) were entered individually. The addition of Population (*p* = .636) and Orientation (*p*= .236) did not significantly improve fit when compared with the baseline model. However, the addition of Motivation yielded a significant improvement in fit when compared with the baseline model (χ^2^=8.83, *p* =.002). Model 5 included fixed effects of Population + Orientation but did not significantly improve fit when compared with Model 4 (*p* = .262). Model 6 included fixed effects of Population + Motivation but did not significantly improve fit when compared with Model 4 (*p*= .445). Model 7 included fixed effects of Motivation + Orientation and did not significantly improve fit when compared with Model 4 (*p* =.068). Model 8 included fixed effects of Population + Motivation + Orientation but did not significantly improve fit when compared with Model 4 (*p* =.146). Model 9 included a fixed effect of Motivation and a two-way interaction between Population x Orientation and did not significantly improve in fit when compared with Model 4 (*p* = .236). Model 10 included a fixed effect of Population with a two-way interaction between Orientation x Motivation which did not significantly improve in fit when compared with Model 4 (*p* = .366). Model 11 included a fixed effect of Orientation with a two-way interaction of Population x Motivation but did not significantly improve fit when compared with Model 4 (*p* = .238). Finally, the three-way interaction (Population x Motivation x Orientation) was entered (Model 12); doing so did not significantly improve fit in comparison to Model 4 (*p* =743). Thus, Model 4, containing a fixed effect Motivation alone provided the best fit to the observed data.

***Justifications for Telling the Truth:***

**Model Building Sequences for Justification for Telling the Truth Data Presented in the Supplementary Materials**

***Morally Wrong***

We began with a baseline model containing by-participant, by-scenario, and by-questionnaire-order intercepts with a random slope of motivation per participant and orientation x population slopes per scenario. Fixed effects of Population (Model 2), Orientation (Model 3) and Motivation (Model 4) were entered individually. The addition of Population (*p* = .104) and Orientation (*p*= .145) and Motivation (*p* =.406) did not significantly improve fit when compared with the baseline model. Model 5 included fixed effects of Population + Orientation and yielded a significant improvement from the baseline model (χ^2^= 8.31, *p* =.039). Model 6 included fixed effects of Population + Motivation but did not differ significantly from the baseline model (*p*= .151). Model 7 included fixed effects of Motivation + Orientation and did not significantly differ from the baseline model (*p* =.267). Model 8 included fixed effects of Population + Motivation + Orientation but did not significantly improve fit when compared with Model 5 (*p* =.901). Model 9 included a fixed effect of Motivation and a two-way interaction between Population x Orientation and did not significantly improve in fit when compared with Model 5 (*p* = .759). Model 10 included a fixed effect of Population with a two-way interaction between Orientation x Motivation but did not yield a significant improvement in fit when compared with Model 5 (*p* = .093). Model 11 included a fixed effect of Orientation with a two-way interaction of Population x Motivation but did not significantly improve fit when compared with Model 5(*p* = .969). Finally, the three-way interaction (Population x Motivation x Orientation) was entered (Model 12); doing so did not significantly improve fit in comparison to Model 5 (*p* =.472). Thus, Model 5, containing a fixed effect of population and orientation, provided the best fit to the observed data.

***Not Believed:***

We began with a baseline model containing by-participant, by-scenario, and by-questionnaire-order intercepts with a random slope of motivation per participant and orientation x population slopes per scenario. Fixed effects of Population (Model 2), Orientation (Model 3) and Motivation (Model 4) were entered individually. The addition of Population (*p* = .402), Orientation (*p*= .077), and Motivation (*p*= .126) did not significantly improve fit. Model 5 included fixed effects of Population + Orientation but did not significantly differ from baseline (*p* =.124). Model 6 included fixed effects of Population + Motivation but did not differ significantly from baseline (*p*= .196). Model 7 included fixed effects of Motivation + Orientation and yielded a significant improvement in fit when compared with baseline (χ^2^ = 9.10, *p* =.028). Model 8 included fixed effects of Population + Motivation + Orientation but did not yield a significant improvement in fit when compared with Model 7 (*p* = .365). Model 9 included a fixed effect of Motivation and a two-way interaction between Population x Orientation but did not significantly improve fit when compared with Model 7 (*p* = .117). Model 10 included a fixed effect of Population with a two-way interaction between Orientation x Motivation but did not significantly improve fit when compared with Model 8 (*p* = .678). Model 11 included a fixed effect of Orientation with a two-way interaction of Population x Motivation but did not significantly improve fit when compared with Model 7 (*p* = .593). Finally, the three-way interaction (Population x Motivation x Orientation) was entered (Model 12); doing so did not significantly improve fit in comparison to Model 7 (*p* =.080). Thus, Model 7, containing a fixed effect of Motivation and Orientation provided the best fit to the observed data.

***Feel Bad:***

We began with a baseline model containing by-participant, by-scenario, and by-questionnaire-order intercepts with a random slope of motivation per participant and orientation x population slopes per scenario. Fixed effects of Population (Model 2), Orientation (Model 3) and Motivation (Model 4) were entered individually. The addition of Population (*p* = .323), Orientation (*p*= .114), and Motivation (*p*= .197) did not significantly improve fit beyond baseline. Model 5 included fixed effects of Population + Orientation but did not significantly differ from baseline (*p* =.207). Model 6 included fixed effects of Population + Motivation but did not differ significantly from baseline (*p*= .317). Model 7 included fixed effects of Motivation + Orientation but did not significantly improve fit when compared with baseline (*p* =.172). Model 8 included fixed effects of Population + Motivation + Orientation but did not significantly improve fit when compared with baseline (*p* =. 272). Model 9 included a fixed effect of Motivation and a two-way interaction between Population x Orientation but did not significantly improve fit when compared with baseline (*p* = .327). Model 10 included a fixed effect of Population with a two-way interaction between Orientation x Motivation but did not significantly improve fit when compared with baseline (*p* = .085). Model 11 included a fixed effect of Orientation with a two-way interaction of Population x Motivation but did not significantly improve fit when compared with baseline (*p* = .154). Finally, the three-way interaction (Population x Motivation x Orientation) was entered (Model 12); doing so did not significantly improve fit in comparison to baseline (*p* =.197). Thus, no model significantly improved fit beyond baseline suggesting neither population, motivation, or orientation predicted the likelihood of participants telling the truth because lying would make them feel bad.

***Data Dictionary***

| Variable Name | Variable Description |
| --- | --- |
| ***Demographics*** |  |
| Ppt_no | The participant number used to anonymise data. |
| Gender | The gender that each participant identified as (male, female, third gender, non-binary, other) |
| Age | Age in years |
| Population | Which population the participant identified as belonging to- non-autistic or autistic |
| Native language | The native language of participants |
| DKEF_Total | The total summed score participants achieved on the DKEF Towers Task |
| DKEF_1^ST^_move | The average number of seconds it took participants to make their 1^st^ move in the DKEF Towers Task |
| DKEF_Time_Per_Move | The average number of seconds between each move participants made on the DKEF Towers Task |
| Vocab | The total score participants achieved on the Vocabulary sub-test of the Abbreviated Weschler Intelligence Scale |
| Similarities | The total score participants achieved on the Similarities sub-test of the Abbreviated Weschler Intelligence Scale |
| Block | The total score participants achieved on the Block Design sub-test of the Abbreviated Weschler Intelligence Scale |
| Matrix | The total score participants achieved on the Matrix sub-test of the Abbreviated Weschler Intelligence Scale |
| Verbal_IQ | The summed total of scores for the Vocab and Similarities variables |
| Practical_IQ | The summed total of scores for the Block and Matrix variables |
| AQ_Total | The summed total of scores for participants completing the Autism Quotient |
| ***General Lying Data*** |  |
|  |  |
| Often_Lie | Scores correlate to frequency with which participants report they lie in general |
| Guilty_General | How guilty participants usually feel when lying in general (1-7) |
| Believed_General | How confident participants are that they would be believed when lying in general (1-7) |
| Difficult_General | How difficult participants find lying in general (1-7) |
| FamilyFace | Frequency of lies told to a family member face-to-face in the past 24 hours |
| FamilyPhoneEmailWriting | Frequency of lies told to a family members over the phone/via email/via writing in the past 24 hours |
| FriendFace | Frequency of lies told to a friend face-to-face in the past 24 hours |
| FriendPhoneEmailWriting | Frequency of lies told to a friend over the phone /via email/via writing in the past 24 hours |
| WorkSchoolFace | Frequency of lies told to a school friend/work colleague face-to-face in the past 24 hours |
| WorkSchoolPhoneEmailWriting | Frequency of lies told to a school friend/work colleague over the phone/via email/via writing in the past 24 hours |
| DontKnowFace | Frequency of lies told to people they barely know face-to-face in the past 24 hours |
| DontKnowPhoneEmailWriting | Frequency of lies told to people they barely know over the phone/via email/via writing in the past 24 hours |
| StrangersFace | Frequency of lies told to a stranger face-to-face over the past 24 hours |
| StrangersPhoneEmailWriting | Frequency of lies told to a stranger over the phone/via email/via writing over the past 24 hours |
| LargeGroupsFace | Frequency of lies told to a large group face-to-face over the past 24 hours |
| LargeGroupPhoneEmailWriting | Frequency of lies told to a large group over the phone/via email/via writing over the past 24 hours |
| 24_Hour_total | The total frequencies of lies told in the past 24 hours |
| TotalFace | The total frequency of lies told face-to-face over the past 24 hours |
| TotalPhoneEmailWriting | The total frequency of lies told over the phone/via email/via writing over the past 24 hours |
| TotalFamily | The total frequency of lies told to family members over the past 24 hours |
| TotalFriend | The total frequency of lies told to a friend over the past 24 hours |
| TotalWorkSchool | Total frequency of lies told to a work colleague/school friend over the past 24 hours |
| TotalDon’tReallyKnow | Total frequency of lies told to people they don’t really know over the past 24 hours |
| TotalStranger | Total frequency of lies told to strangers over the past 24 hours |
| TotalLargeGroup | Total frequency of lies told to a large group over the past 24 hours |
| ***Lie Scenario Data*** |  |
| Questionnaire_Version | Which version of the questionnaire the participant completed (1,2 or 3) |
| Motivation | Whether the scenario had a protective or beneficial motivation |
| Scenario | The title of the hypothetical scenario presented |
| Orientation | Whether the scenario had a self, beneficial or pareto orientation |
| Honest_or_lie | Whether the participant selected to lie or tell the truth in the scenario |
| Lie1_honest0 | Veracity quantified- if they would lie = 1, if they would tell the truth = 0 |
| Guilty-scenario | How guilty participants would feel lying in the scenario (1-7) |
| Believed-scenario | Participants reports of how confident they are that they would be believed when lying (1-7) |
| Difficult-scenario | Participants reports of how difficult they would find telling the lie (1-7) |
| Not_Believed | Participants reports of choosing not to lie due to fear of not being believed (0,1) |
| Morally_Wrong | Participants reports of choosing not to lie as the lie would have been morally wrong (0,1) |
| Feel_Bad | Participants reports of choosing not to lie as doing so would make them feel bad (0,1) |
| Other | Participants could select other if none of the other 3 options reflected their decision to tell the truth |
| Why_Truth | If participants did select other, they were provided with the opportunity to write their reason in an open text box |
| Guilty_Truth | How guilty participants would feel after telling the truth (1-7) |
| Happy | How happy participants would feel after telling the truth (1-7) |
| Proud | How proud participants would feel after telling the truth (1-7) |
| Relieved | How relieved participants would feel after telling the truth (1-7) |
| Relaxed | How relaxed participants would feel after telling the truth (1-7) |
| Other | If participants believed they would feel a different emotion they could select other |
| How_truth__feel_other | If participants did select other they were provided with an open text box in which they could write any other emotions they would experience |
